# Training for generalization in Theory of Mind: a study with older adults

**DOI:** 10.3389/fpsyg.2015.01123

**Published:** 2015-08-04

**Authors:** Elena Cavallini, Federica Bianco, Sara Bottiroli, Alessia Rosi, Tomaso Vecchi, Serena Lecce

**Affiliations:** ^1^Department of Brain and Behavioral Sciences, University of Pavia, Pavia, Italy; ^2^Headache Science Centre, National Neurological Institute C. Mondino, Pavia, Italy

**Keywords:** Theory of mind, training, older adults, generalization effect, mentalizing ability

## Abstract

Theory of Mind (ToM) refers to the ability to attribute independent mental states to self and others in order to explain and predict social behavior. Recent research in this area has shown a decline in ToM abilities associated with normal aging that is of a moderate magnitude or greater. Very few studies have investigated whether it is possible to improve older adults’ ToM abilities. The present study was designed to address this gap in the literature by evaluating the impact of a ToM training on practiced and transfer tasks. We provided older adults with a variety of activities designed to facilitate the generalization of benefits to other ToM-demanding tasks. Participants were 63 healthy older adults, native Italian speakers (*M*_*age*_ = 71.44, SD = 5.24, age range: 63–81 years). Participants were randomly assigned to one of two groups: the ToM training (age range: 63–81 years) and the physical-conversation training (age range: 64–81 years). Training effects were measured using the strange stories (practiced task) and the animation task (transfer task). Results revealed the efficacy of the training in producing improvements on practiced but also on transfer tasks.

## Introduction

Theory of Mind (ToM) refers to the ability to attribute independent mental states to self and others in order to explain and predict social behavior (Carruthers and Smith, 1996; Baron-Cohen et al., 2000). It is a fundamental skill used throughout the life span, with important implications for social communication abilities and social relationships (Henry et al., 2013). Recent research in this area has shown a decline in ToM abilities associated with normal aging that is of a moderate magnitude or greater and that can only be partially attributed to a more general decline in cognitive skills associated with making inferences (for a review, see Henry et al., 2013).

Despite the fact that many studies have shown an age-related decline in mental state understanding and have highlighted the importance of ToM for social relationships (Shamay-Tsoory and Aharon-Peretz, 2007; Krach et al., 2010), to date only one study has tested the possibility to train older adults on ToM. It is a study by Lecce et al. (2015) on a non-clinical sample. Older adults (age range: 58–85 years) were assigned to one of three training conditions: ToM-conversation, physical-conversation and social-contact. In the ToM-conversation condition, older adults took part in guided conversations about mental states. In the physical-conversation training, subjects took part in conversations that focused on physical rather than mental inferences. The ToM- and the physical-conversation trainings were closely matched in length and structure, and both used short narratives as prompts. Finally, in the social-contact group, participants took part in general conversations about aging, without a specific focus on mental states inferences. Results showed that the ToM training group increased its ToM skills significantly more than the other two control groups, supporting the view that advanced ToM skills may be enhanced in healthy aging through mental states conversations.

Although interesting, Lecce et al. (2015) study is limited in that the training focused on a single group of tasks and adopted a verbal modality only. Is also paid little attention to the application of ToM to real life contexts in which people receive information from a combination of sources and modalities. For example, a recent model proposed by Achim et al. (2013) called the eight sources of information framework (8-SIF), posits that mentalizing in real life requires to take into account eight different sources of information as they emerge from the cross of the following main sources: (1) immediate, (2) memory, (3) agent, and (4) context. Some of these information are readily available through our senses, whereas others are less explicit and are stored in memory. Notably, the discrepancies between mentalizing activated in Lecce’s study and used in real life context are relevant for two main reasons. First, ToM is a multicomponential ability involving several processes at a number of levels, from perceptual to conceptual (Castelli et al., 2000; Stone and Gerrans, 2006). Second, the use of a single type of task limits the generalization of the program’s effects. Given that the most desirable outcome of a cognitive intervention is the possibility to transfer the benefit to material that is not practiced during the training, research should be focused on designing training programs able to produce generalization to several components of ToM. This issue represents the focus of the present study. Generally speaking, the transfer effect is related to the existence of brain plasticity. The maintenance of a certain grade of plasticity in aging (e.g., Greenwood, 2007) is demonstrated by the effectiveness of cognitive interventions aimed to preserve an adequate cognitive functioning, limiting the normal decline of several functions, such as memory (e.g., Cavallini et al., 2010; Bottiroli et al., 2013) and reasoning (e.g., Anand et al., 2011). Given the possibilities to change cognitive performance in aging, many approaches to trainings have been attempted to promote transfer (e.g., Cavallini et al., 2003a,b; Jennings et al., 2005; Lustig and Flegal, 2008).

Existing studies on this issue have highlighted the importance of three features of the training (e.g., Zelinski, 2009; Cavallini et al., 2010; McDaniel and Bugg, 2012; Bottiroli et al., 2013): repetition, variability of tasks, and a learner-oriented approach. Zelinski (2009), in the field of cognitive interventions, proposed that extended practice training involving extensive repetition of tasks produces transfer in multiple domains in aging studies. It is a bottom up approach that leads to improvement of basic cognitive skills. Since extended practice approach tends to limit the use of domain-specific strategies, it has greater potential than programs based on strategy learning in producing transfer to untrained tasks. This is because extended practice approach stimulates the underlying cognitive skills of the target ability. This approach is found to be successful with respect to executive functions (Bherer et al., 2005; Tang and Posner, 2009). For instance, interventions based on practicing attention and inhibition have been found to be successful in increasing working memory skills (Borella et al., 2013). Regarding the second feature of the training (i.e., variability of tasks), McDaniel and Bugg (2012) have suggested that increasing the variation that is experienced during the course of training, at the level of stimuli and tasks, is crucial to improve transfer. Indeed, the diversity of tasks increases the chance that training exercises will produce desired gains. In the best case scenario, the tasks could contribute to performance improvement in an additive fashion, and thus yield large transfer effects. As far as the third feature is concerned (i.e., a learner oriented approach), Cavallini et al. (2010) and Bottiroli et al. (2013) have highlighted the importance of adopting a learner-oriented approach based on strategy adaptation in order to better encourage transfer effects. They recommend treating older adults as active partners in attempting to achieve generalization in untrained tasks (learner-oriented approach). The core method of this approach is to involve older adults not only in practice on different tasks, but also in analyzing the features of tasks and in discussing how strategies could be applied to new materials (strategy adaptation). This approach is predicated on the premise that spontaneous generalization rarely occurs. Indeed, older adults may not realize that abilities/strategies trained within one task context can be modified or adapted to slightly different material and contexts (Hertzog and Dunlosky, 2012). An active involvement of older adults in the training would permit them to overcome this limitation.

### The ToM Training

The studies cited above (Cavallini et al., 2010; McDaniel and Bugg, 2012; Bottiroli et al., 2013) provide important insights into the principles of effective ToM training. Therefore, in designing our intervention, we treated participants as active partners in the transfer process, making them aware that the various ToM tasks have different characteristics, rely on different modalities (i.e., verbal visual, static, and dynamic), and are based on a specific combination of agent- and context-related information. Crucially, in our training, we provided participants with practice on a variety of ToM tasks and made them reflect that using ToM in an appropriate way requires the attribution of mental states to a character/person given a specific context. We designed a set of activities using an array of materials and modalities to stimulate advanced ToM reasoning. All these activities required the interpretation of others’ mental states. More precisely, we used stories, oral stimuli, and static images. These activities were followed by guided group conversations in which the experimenter kept to a series of pre-developed questions as prompts and made sure that all people took part in the conversation, discussing their points of view. This is because there is strong agreement in the literature that mental states conversations predict later ToM skills (Appleton and Reddy, 1996; Peterson and Slaughter, 2003; Ensor and Hughes, 2008; Ornaghi et al., 2011; Lecce et al., 2014b). Notably, in these conversations we paid special attention to adopt a dynamic approach in order to make people reflect that mental states are transitory and can change over time. Thus, in our training program we presented people with a variety of complex social situations (e.g., misunderstanding, faux-pas) and asked them to solve the social problems of each social scenario. To this end, after having presented the social scenario, we asked our participants the following question: *What could you do or say in order to resolve the problem?* We adopted this strategy because trying to find a solution is a useful methodology to actively involve participants in the training (Bottiroli et al., 2013). Older adults were also asked to imagine a personal situation similar to that reported in the story presented by the experimenter and describe how he/she would have resolved it. Requiring participants to think of individual experience makes the task more meaningful. It also represents a method to help participants to realize that the skills that they have been working on during the training can be transferred to everyday life.

Notably, in designing our training we followed the SAFE norms that are known to maximize the training effects (Durlak et al., 2011). According to this, a training program should be sequenced, active, focused, and explicit. In our intervention we connected and coordinated sets of activities respecting increasing levels of difficulty (i.e., Sequenced). We gave participants the opportunity to act on the material, since it is well documented that practice is a necessary condition for skill acquisition (Zelinski, 2009; McDaniel and Bugg, 2012; i.e., Active). We dedicated enough time and attention to every ToM task for learning to occur (i.e., Focus). Finally, we explicitly stated the learning aims in order to make older adults aware of what they were expected to learn in terms of mentalizing abilities and that skills learnt during the training can be used with other materials and in everyday life (i.e., Explicit).

### The Current Study

The aim of the present study was to test the efficacy of a new ToM training designed to promote mental states understanding and to investigate the generalization to a far ToM transfer task. Participants were randomly assigned to one of two groups: the ToM training and the physical-conversation training. As previously stated, in the ToM training participants were involved in several tasks and conversations on mental states. In the physical-conversation training, older adults practiced and discussed materials about physical occurrences with a trainer. Participants were involved in inferential discussion without using mental state expressions. To evaluate transfer effects, we selected a non-verbal ToM task, the animation task (Abell et al., 2000; Castelli et al., 2000) that selectively evokes mental state attribution by its motion properties. The use of a task that differs in structure (involving humans vs. geometric shapes) and modality (verbal static vs. visual dynamic) from those used in the training is crucial as it allows researchers to test if the training simply fostered the set of skills necessary to solve the practiced task or produced a genuine change in ToM understanding. The animation task (Abell et al., 2000; Castelli et al., 2000) can be considered as a far transfer task as cues for mental state attributions are restricted to pure movements and interactions without any vocal or facial expression prompts. This task has been successfully used in clinical (Abell et al., 2000; Salter et al., 2008; White et al., 2011), neurological (Castelli et al., 2000, 2002; Bird et al., 2004), and developmental studies (Campbell et al., 2006; Moriguchi et al., 2007). Overall, these studies have shown that these animations successfully detect mentalizing difficulties in individuals with autism (Abell et al., 2000), schizophrenia (Russell et al., 2006), and activate brain regions implicated in mental state understanding (Castelli et al., 2000). The rationale for expecting an improvement in this far transfer task is that our dynamic approach will foster the accuracy of mental state attributions and the ability to understand the mental states involved in a social situation (Bianco et al., submitted).

## Materials and Methods

### Participants

Participants were a total of 63 healthy older adults, native Italian speakers (*M*_*age*_ = 71.44, SD = 5.24, age range: 63–81 years). They were recruited through the University of Third Age and aggregation centers located in the North of Italy. The inclusion criteria were: no psychiatric or neurological diseases and no cognitive impairment. No tangible incentives (e.g., money or gifts) were given to participate. Participants were randomly assigned to one of two groups: the ToM training (age range: 63–81 years) and the physical-conversation training (age range: 64–81 years). Preliminary separate one-way analyses of variance were performed on background variables to establish the equivalence of the two groups before the training. Groups did not differ significantly in age [*F*(2,62) = 0.00, MSE = 27.86, *p* = 0.98, η^2^ = 0.00], years of education [*F*(2,62) = 0.13, MSE = 9.22, *p* = 0.72, η^2^ = 0.00], or vocabulary [drawn from the Primary Mental Abilities test; Thurstone and Thurstone, 1963; *F*(2,62) = 2.80, MSE = 22.69, *p* = 0.10, η^2^ = 0.04]. Descriptive statistics for age, percentage of males and females, years of education, and vocabulary are shown in Table 1. All participants completed and accepted an informed consent form prior to beginning of the study.

**TABLE 1:** **Participants characteristics of ToM training and physical-conversation training**.

**Participant characteristics**	**ToM training (*n* = 37)**	**Physical-conversation training (*n* = 26)**

Age	71.43 (5.05)	71.46 (5.59)
% Female	86.5	85.5
Years of education	9.43 (2.96)	9.15 (3.15)
Vocabulary	42.81 (4.21)	40.78 (5.46)

Age ranged from 63 to 81 years. Maximum vocabulary score = 50. Scores in parenthesis refers to Standard Deviation.*.1pt

### Measures

#### Primary Mental Abilities Test

At pre-test, verbal skills were assessed through the Vocabulary subtest of the PMA test (Thurstone and Thurstone, 1963). The Vocabulary test was used to gain an objective measure of the participants’ actual level of education and an estimate of their crystallized intelligence. Since it evaluates knowledge of words meaning, subjects were asked to identify the correct synonym of 50 target words by selecting one of four alternatives. Time limit was 8 min. One point was given for each correct answer (range 0–50).

#### Strange Stories Task

The strange stories task (White et al., 2009) was used to assess the efficacy of our training. We administered 10 ToM stories and four Physical stories of the strange stories task at both pre- and post-test. ToM stories assessed the ability to understand people’s mental states in five scenarios involving misunderstanding, double bluff, persuasion, sarcasm, and white lie. The Physical stories assessed the ability to make inferences on physical (rather than mental) events and required the integration of information between sentences and inference from implicit information. The two types of stories were similar in length and complexity. After having read the stories, subjects were asked to answer the test question. Participants were allowed to consult the text until a response was given in order to avoid memory loads. Responses were rated using a three-point scale: 0 for incorrect answers, one for partially correct answers, and two for full and explicit answers (range 0–20 for ToM stories and 0–8 for Physical stories). Two raters independently coded 25% of the responses at pre- and post-test and inter rater agreement was established using Cohen’s Kappa. This agreement was good for both the mental (at pre-test, κ = 0.91; at post-test, κ = 0.79) and the Physical stories (at pre-test, κ = 0.71; at post-test, κ = 0.83).

#### Animation Task

The animation task (Abell et al., 2000; Castelli et al., 2000) was used to measure transfer effects of our training. It evaluates individual’s ability to attribute mental states to animated geometric shapes. We administered four ToM and four Goal-directed animations at both pre- and post-test. All the animations featured two characters, a big red triangle and a small blue triangle, moving about on a framed white background. Each sequence lasted between 34 and 45 s. In the ToM condition, interaction between the two triangles was scripted to imply complex mental states. In one animation, one triangle tried to persuade the other to let it go free; another sequence showed the little triangle surprising the big triangle; the third animation represented the small triangle mocking the big one, and the last one showed the big triangle coaxing the little one out of an enclosure. In the control “Goal-directed” condition, the characters interacted on a simple, behavioral basis. The interactions represented in this condition were reciprocal but did not involve mental states. One sequence showed the smaller triangle following the bigger one, and the remaining three represented the triangles fighting, chasing one another, and dancing together. Animations were presented in a pseudo-random order, counterbalanced within each group. Before starting the task, the experimenter informed the participants that they would be shown eight animations on a computer, with two triangles interacting between themselves. At the end of each animation, participants were asked to describe what happened in the animation. No feedback was given. Answers were coded for the level of intentionality. This score reflects the degree of attribution of mental states to the shapes, ranging from 0 (non-deliberate action), to 5 (deliberate action aimed at affecting another’s mental state; possible score range 0–40). Two raters independently coded 25% of the responses at pre- and post-test and inter rater agreement was established using Cohen’s Kappa. The inter rater agreement was good (at pre-test, κ = 0.87; at post-test, κ = 0.80).

Each response was also assigned to one of three types of descriptions: action, interaction, mentalizing. Any response comprising a simple action statement (no mention of interaction between the triangles, or mental states) was rated as action (e.g., “the triangles are bouncing”). Any response that explicitly mentioned interaction between the triangles (no mention of mental states) was classified as interaction (e.g., “the little triangle touches the big one”). Descriptions that included mental state terms were classified as mentalizing (e.g., “the triangles are happy”).

### Design and Training Procedure

Participants were pre-tested on ToM tasks and on verbal ability. Information about level of education was also gathered during the pre-test. After the pre-test, subjects took part in a training program that consisted of four lessons. At the end of the training program, they were post-tested on ToM tasks to evaluate the effects of the training.

The training program in both conditions was conducted by female trainers. The activities in both groups increased in the level of complexity lesson after lesson and made use of a range of modalities and materials (visual stimuli, audio stimuli, and written stimuli). The activities of the control condition strictly matched those of the experimental condition, but focused on physical rather than mental states inferences. Below, more details for each condition are provided.

A full description of the training materials in each condition is provided in Table 2.

**TABLE 2:** **Description of training lessons by group**.

**Lesson**	**Component**	**Material**	**ToM training**	**Physical-conversation training**
1	Introduction to the main content of the training	Exemplifying stimuli	The trainer presented the aims of the ToM training and were introduced to the nature of inferences on mental states and on its relevance in real life. Subjects practiced with examples of scenarios requiring mental-state inferences and reasoning. Material adapted from Lecce et al. (2014b, 2015).	The trainer presented the aims of the physical-conversation training and were introduced to the nature of inferences on physical states and on its relevance in real life. Subjects practiced with examples of scenarios requiring physical inferences and reasoning. Material adapted from Lecce et al. (2014b, 2015).
2	Visual perspective-taking	Visual stimuli	The trainer presented eight visual stimuli depicting a room where an avatar stood behind a table. Unambiguous (e.g., 8) or ambiguous (e.g., 6) numbers were shown either on the wall or on the table. When the ambiguous numbers were shown on the table the subjects and the avatar perceived the stimulus differently. Participants were required to make a judgment about how the numeral appeared to themselves and how the number appeared to the avatar in the scene. Material adapted from Surtees et al. (2012).	The trainer presented eight visual stimuli depicting a 4 4 grid. Subjects were told the grid was a bookcase. Some slots contained objects and participants were instructed to imagine they had to push certain objects, in order to drop them on the floor. Some slots in the grid were occluded behind so that the object could not be dropped. Critical instructions required the participants to ignore objects in the occluded slots and to push other objects, in order to accomplish the request. Material adapted from Dumontheil et al. (2010).
3	Conceptual perspective-taking	Visual stimuli	The trainer showed a full picture and subsequently a small portion of the same picture. Participants were asked to imagine what two individuals, exposed only to the small portion of the picture, would think the image was. Seven pictures were shown. Material adapted from Lalonde and Chandler (2002).	The trainer showed a complete picture and subsequently a small portion of the same picture. Participants were asked to recollect which part of the big image the small portion depicted. Seven pictures were shown. Material adapted from Lalonde and Chandler (2002).
	Conceptual perspective-taking	Audio stimuli	The trainer presented participants with three very short oral stories, where a misunderstanding between characters occurred. These oral texts were read by the trainer and were based on Italian idiomatic expressions containing ambiguous words with double meaning. The contextual information of these stories was kept to the minimum. Participants answered a series of questions about: - character’s beliefs and points of view; - what the main character could do or say in order to resolve the problem.	The trainer presented participants with three very short oral stories about physical phenomena. Participants answered a series of questions about: - specific facts of the story or details; - the physical event not explicitly mentioned in the text (inference). Material adapted from Lecce et al. (2014b, 2015).
	Conceptual perspective-taking	Written stimuli	The trainer presented seven mental state stories similar to those of the revised strange stories task (White et al., 2009). The stories referred to complex social situations. Participants answered a series of questions about: - the main character’s mental state; - one character’s belief about the other characters' mental state; - mental state underlying social behavior; - what the main character could do/say in order to resolve the problem. Older adults were asked to imagine a personal situation similar to that reported in the story and describe how he/she would have resolved it.	The trainer presented seven stories about physical phenomena similar to those of the revised strange stories task (White et al., 2009). Participants answered a series of questions about: - specific facts of the story or details presented in the text; - the physical event not explicitly mentioned in the text (inference).
4	Real-life perspective-taking	Audio stimuli	The trainer presented, through audio stimuli, three pieces of real-life conversations, each involving two human characters. Conversations were rich in mental states terms and referred to ambiguous and complex social situations (e.g., misunderstanding, sarcasm). After having listened to each conversation, participants answered a series of questions about: - character’s beliefs and points of view; - mental states involved; - what the main character could do or say in order to resolve the problem. Older adults were asked to imagine a personal situation similar to that reported in the conversation and describe how he/she would have resolved it.	The trainer presented, through audio stimuli, one oral description about non-mental phenomena. After having listened to the stimulus, participants answered a series of questions about what they had just heard.
	Real-life perspective-taking	Written stimuli	The trainer presented a short portion of a novel (Coelho, 1998, p. 88) to participants. Participants answered a series of questions about: - character’s mental states and points of view; - mental-states lexicon involved. Older adults were asked to imagine a personal situation similar to that reported in the portion of the novel and describe how he/she would have resolved it.	The trainer presented two written riddles to participants. Riddles were ecological so that they were similar to the ones in newspapers. In order to correctly answer, details of the text needed to be linked and an inference beyond explicit information was required.

#### ToM Training

The ToM training program of the present study was based on that of Lecce et al. (2015) and, thus, made extensive use of the mental states narratives that were presented in each of the four lessons. In addition to this material, the present training program proposed a set of activities of increasing difficulty, that required the understanding that people can have different perspectives of the same reality. In the first lesson, people were explicitly told the main aim of the training program and were familiarized with the cognitive activities and processes involved in the intervention. The second lesson focused on visual perspective-taking, the third lesson focused on conceptual perspective-taking, and the fourth lesson on real-life perspective taking. The visual-perspective-taking tasks made participants reflect on the fact that the same stimulus (e.g., a number) may be perceived differently by asking them to view it from the other’s perspective. The conceptual perspective- taking activities were designed to help participants to recognize that two people might have different desires, beliefs, or access to knowledge. These activities were presented using three modalities: visual, written, and audio. Finally, the real-life perspective-taking activities were designed to be more complex tasks as they require people to consider more sources of information at the same time (Achim et al., 2013). These information regarded both the agents and context and could be found in the immediate scenario and in memory. These real-life perspective-taking activities, thus, assessed the ramifications of such perspective taking in complex, contextualized social scenarios. Participants were presented with real-life scenarios and asked to infer the beliefs and desires of human characters to explain their behaviors. These activities were presented using the oral and written formats.

After each single item of each group of tasks, participants were asked to individually answer a series of questions in order to elicit a complete and explicit understanding of the mental states and behavior of characters. In order to promote the *dynamic nature* of mental states, participants were asked how they could resolve social ToM situations. Older adults were also asked to imagine a personal situation similar to that reported in the task and describe how they would have resolved it. In each lesson, individual practice was followed by group discussions led by the trainer. Crucially, the trainer used mental state expressions, and made frequent use of positive and corrective feedback.

#### Physical-Conversation Training

The physical-conversation training was designed to be closely matched to those of the experimental condition. It had the same structure and the same length of the ToM training. The main difference was that the stimuli and the content of the stories that were discussed in the training were about physical, not mental occurrences. Participants practiced on a range of materials but without a focus on mental states. During the training, participants were asked to make inferences on physical, rather than mental, features of the stimuli. In each lesson, individual practice was followed by group discussions led by the trainer. The trainer encouraged participants to take part in the group conversations, provided feedback but, crucially, made no use of mental state expressions.

## Results

### Preliminary Analysis

Before examining the training effects, we compared individuals’ performance at the pre-test on the various tasks by running a series of *t*-tests on the percentage of correct responses. The comparison between the two ToM tasks showed that performance on the ToM stories (*M* = 61.17, SD = 14.22) was significantly higher than that on the ToM animations [*M* = 54.17, SD = 15.46; *t*(59) = 2.83, *p* = 0.01], revealing the different level of complexity of these two tasks. For the animation task, results showed a higher level of intentionality scores in the ToM (*M* = 53.93, SD = 15.22) than in the goal-directed animations [*M* = 43.01, SD = 12.36; *t*(62) = 5.95, *p* < 0.001].

In addition, to establish the equivalence of the two training groups at the pre-test, preliminary separate one-way analyses of variance were performed on ToM tasks. Results showed that groups did not differ in their pre-test scores on ToM stories [*F*(2,58) = 1.26, *p* = 0.27, η^2^ = 0.02], physical stories [*F*(2,60) = 0.00, *p* = 0.95, η^2^ = 0.00], ToM animations [*F*(2,61) = 2.51, *p* = 0.18, η^2^ = 0.04], and goal-directed animations [*F*(2,61) = 0.14, *p* = 0.71, η^2^ = 0.00; Table 3]. Similar results were obtained when we considered the frequency of participants’ answers on each type of description of the ToM animations: action, interaction, and mentalizing. Indeed, at the pre-test, groups did not differ either in the interaction [*F*(2,61) = 0.14, *p* = 0.71, η^2^ = 0.00] or in mentalizing [*F*(2,61) = 1.76, *p* = 0.19, η^2^ = 0.03]. The only significant difference was found on pre-test scores in the action description [*F*(2,61) = 5.04, *p* = 0.03, η^2^ = 0.08], where the physical-conversation training group reported a higher score than the ToM training group.

**TABLE 3:** **Means value and standard deviations for ToM task performance as a function of Group condition (ToM and physical-conversation groups) and Time (pre- and post-test)**.

	**ToM training**		**Physical-conversation training**	
	**Pre**	**Post**	**Pre**	**Post**
Strange stories	59.42 (14.89)	79.62 (12.70)	63.60 (13.11)	75.56 (17.03)
Physical stories	65.62 (16.48)	68.05 (19.24)	65.86 (13.94)	67.79 (20.36)
ToM animation Intentionality	56.49 (12.68)	64.00 (12.17)	50.38 (17.88)	54.00 (14.36)
Action descriptions	0.24 (0.49)	0.17 (0.57)	0.73 (1.18)	0.36 (0.86)
Interaction descriptions	2.67 (0.91)	2.28 (1.07)	2.46 (1.03)	2.68 (0.90)
Mentalizing descriptions	1.08 (1.01)	1.54 (1.07)	0.77 (0.76)	0.96 (0.67)
Goal-directed animation Intentionality	43.51 (10.46)	45.75 (10.38)	42.31 (14.85)	44.20 (12.72)

Scores in parenthesis refers to Standard Deviation.

### ToM Training Effects

In order to analyze the effect of the training, a mixed-design ANOVA was conducted on each task, with time (pre- and post-test) as the within-subjects factor and training group (ToM and physical-conversation) as the between-subjects factor. Since on pre-test score of ToM animations the two groups tended to differ, although not significantly, we adopted a rigorous approach and ran ANCOVA on gain scores controlling for performance at pre-test. Gain scores were computed by subtracting pre-test scores from the corresponding score on the post-test score. Finally, we were also interested in examining on which type of description (action, interaction, and mentalizing) the ToM- vs. physical-conversation training had an effect. In order to answer this question we ran a mixed-design 2 (time) × 2 (training group) × 3 (type of description) ANOVA.

#### ToM Practiced Task

For ToM stories, results showed a significant main effect of time, *F*(1,57) = 55.21, MSE = 126.78, *p* < 0.001, η^2^ = 0.49 and a significant time by group interaction, *F*(1,57) = 5.79, MSE = 126.78, *p* = 0.02, η^2^ = 0.09. An inspection of the scores at pre- and post-test showed that the ToM group improved more than the physical-conversation group.

For Physical stories, neither the main effect of time, *F*(1,59) = 0.52, MSE = 236.17, *p* = 0.47, η^2^ = 0.01, nor the time by group interaction, *F*(1,59) = 0.00, MSE = 236.17, *p* = 0.97, η^2^ = 0.00, were significant.

#### ToM Transfer Task

For ToM animations, results revealed a significant main effect of time, *F*(1,58) = 6.25, MSE = 118.09, *p* = 0.01, η^2^ = 0.10. The time by group interaction was not significant, *F*(1,58) = 0.83, MSE = 118.09, *p* = 0.37, η^2^ = 0.01. Since on pre-test score of ToM animations the two groups tended to differ, although not significantly, we ran an ANCOVA with gains as the dependent variable and pre-test performance as covariate. Although the effect of the covariate variable was significant *F*(1,57) = 37.65, MSE = 5.79, *p* < 0.001, η^2^ = 0.40, results revealed that the ToM training group improved more than the physical-conversation training group, *F*(1,57) = 5.74, MSE = 5.79, *p* = 0.02, η^2^ = 0.09.

Results from the mixed-design 2 (time) × 2 (training group) × 3 (type of description) ANOVA showed that the time by group by type of description interaction approached significance, *F*(2,116) = 2.80, MSE = 0.84, *p* = 0.06, η^2^ = 0.05. In order to better describe this interaction, we conducted three separate ANOVAs for the action, interaction, and mentalizing gain scores, respectively. Since groups differed on the action and (marginally) on mentalizing at pre-test, we covaried controlling for these variables.

For gains in action descriptions, there were no significant differences between groups, *F*(1,58) = 0.05, MSE = 0.45, *p* = 0.82, η^2^ = 0.001; note that the pre-test action score was a significant covariate, *F*(1,58) = 50.13, MSE = 22.41, *p* < 0.001, η^2^ = 0.47. For gains in interaction descriptions, our analysis revealed that the ToM training group decreased the frequency of use of interaction descriptions in response to ToM animations, whereas the physical-conversation group increased it, *F*(1,58) = 4.88, MSE = 1.22, *p* = 0.03, η^2^ = 0.08. For mentalizing descriptions, although pre-test score was significant as covariate, *F*(1,58) = 2.34, MSE = 0.79, *p* < 0.001, η^2^ = 0.34, the ToM training group showed higher gains in the frequency of use of mentalizing descriptions in response to ToM animations than the physical-conversation group, *F*(1,58) = 4.41, MSE = 0.79, *p* = 0.04, η^2^ = 0.07. These data are crucial as they have revealed that the ToM training program influences the frequency of use of mentalizing descriptions to describe the animations.

For Goal-directed animations, the effect of time, *F*(1,58) = 1.24, MSE = 87.79, *p* = 0.27, η^2^ = 0.02, and of the time by group interaction were not significant, *F*(1,58) = 0.09, MSE = 87.79, *p* = 0.76, η^2^ = 0.00.

## Discussion

This study evaluated the effects of a ToM training program based on conversations about mental states and on a dynamic approach in healthy older adults. Results of the present study are promising as they indicate that our ToM training is effective in promoting ToM performances and transfer. Indeed, the ToM training advantage was evident in both the practiced and the transfer ToM tasks. The results from the strange stories (practiced ToM task) confirmed findings of our previous study (Lecce et al., 2015). The positive results in the animation task (transfer ToM tasks) are particularly relevant given that this ToM task strongly differs from those used during the training. This task makes use of non-verbal stimuli with geometric shapes as protagonists, instead of human beings. Here, it should also be noted that the animation task does not present any relevant contextual information that can help individuals in making inferences, as participants are deprived of any external and social cues. These characteristics make the animation task a challenging measure of ToM and ensure that our transfer effect produces a promising result. Interestingly, we coded participants answers to consider the type of description they used to illustrate the content of ToM animations. This, we believe, was crucial for at least two reasons. Firstly, it allowed us to see whether the animations evoked differential responses from people in the two training conditions. Secondly, it allowed us to examine in detail the nature of the change produced by our training program. Notably, our results revealed that at the post-test participants in the ToM training decreased the use of interaction descriptions and improved that of mentalizing description. These results revealed that our training program helps participants to make a transition from interaction to mentalizing description. Thus, our training was effective in promoting older adults’ ability to attribute mental states to non-verbal and online stimuli in ToM situations. Here, it should also be noted that participants in the control group increased their interaction descriptions after the training. This result was expected and can be explained if we consider that the activities of the control group were focused on physical inferences and were directed to make people reflect on the physical features of a given situation.

An interesting issue, here, regards the mechanisms that underlie the positive effects of our training. Transfer can arise from a variety of reasons and the design of our study did not allow us to fully examine this issue and to answer the question of what did participants learn from our ToM training and how they transfer this acquisition to a new task. However, some speculative explanations can be proposed. We believe that our training program helped older adults to become more sensitive to and aware of mental states that guide social behaviors. In addition, our training is likely to have strengthened older people’s ability in making appropriate context-sensitive inferences to detect relevant information in social situations, and to construct appropriate models of complex social interactions. This last seems to be a mechanism that is particularly crucial in driving ToM advanced development beyond early childhood (Bianco et al., submitted).

We also argue that the benefits of this intervention, in terms of transfer effect, derive, at least in part, from the dynamic approach we used. Indeed, during our training participants were asked not only to analyze and reflect on the different mental features of complex social situations (such as misunderstandings and double bluffs), but also on how they could resolve these situations. Making older adults reflect on the fact that mental states are not static but can change over time is, we think, a prerequisite for obtaining a change in ToM. In addition to the dynamic nature of the activities and to the learner oriented approach, the ToM training was unique in that it made extensive use of conversations on mental states, that is likely to have played a role in improving the efficacy of the training. Theoretical (Nelson, 2005) and empirical findings support the view that conversations on mental states help individuals to improve their awareness that others have different points of view on the same situation and make them able to use their training experience to reflect on the mind of self and others (Lecce et al., 2015). This is important for older adults who show a reduction in social network size (Antonucci, 2001), with a consequent decrease in experience of daily social interactions (Wang and Su, 2013). Crucially, the ToM training group reported a better ToM performance than the matched active control group that made use of conversations on physical, rather than mental, states. This indicates that what matters in terms of ToM development are not the general features of social conversations, but their mental nature. The same conclusion can be drawn for preschoolers (Lecce et al., 2014a) and school aged children (Lecce et al., 2014b). This result is, we believe, interesting as it suggests that the mechanisms involved in the development/improvement of the ToM abilities can be similar throughout the life span.

Our results are certainly important from both a theoretical and a practical point of view. Theoretically, they provide evidence that not only cognitive abilities (such as memory) can be improved in aging, but also that socio-cognitive skills are sensitive to interventions, confirming the plasticity of older people (Greenwood, 2007). In relation to this issue, Rosi et al. (2015) have recently conducted a study comparing old (range: 60–69 years) to old–old (range: 70–85 years) people’s performance on ToM tasks after a ToM training. Interestingly, they found that not only the old, but also the old–old participants increased ToM performance after the training, suggesting a similar level of plasticity in the two age groups. In addition, we believe that our data are theoretically interesting as they fit with the idea that ToM skills cannot be totally explained by general cognitive skills, such as executive function. Indeed, our training poses few emphasis on inhibition, shifting, and working memory. So, the positive effects that we found speak to the idea that executive function, although important, are only one of the possible mechanisms underlying ToM.

From a more practical point of view, our results can be interesting for the treatment of those clinical age-related conditions associated with a ToM deficit, such as Parkinson or Alzheimer diseases (for a review, see Kemp et al., 2012). Hence, they open a new door for ToM intervention research and encourage new training efforts to hone ToM approaches for training. The next step, we believe, will be to verify whether our ToM training, or adapted versions of it, is also effective in improving ToM performance of older adults affected by neurodegenerative diseases.

Some limitations of the current study should also be mentioned. The first concerns the participants of our study. In the training we involved older adults belonging to the University of Third Age and aggregation centers. This may have maximized benefits of our training as these participants were motivated in taking part in the lessons and had several opportunities to use ToM skills. Future studies should therefore be conducted with other older adults selected from the general population who are less involved in social relationships. The second limitation regards the design of our study. We focused mainly on the change in performance from pre-test to post-test, and we did not consider what variables could be responsible for the ToM improvement. In the future, cognitive (such as executive functions and problem solving) and social variables (such as quantity and quality of close social relationships) should be measured and considered as possible predictors of the success of a training.

Future research should also examine the social consequences of improvements in ToM. This is a very interesting issue as for older adults social relationships are crucial and old age is characterized by a change in social relationships (Carstensen et al., 2003). We also hope that our work will stimulate research into the specific processes that underpin older adults’ progress in reasoning about mental states.

In conclusion, the current findings extend the existing literature on ToM interventions for older adults by showing that our ToM training program was effective in stimulating gains in ToM tasks that were not practiced during the training and that differed in several ways from those used in the training. Our results illustrate the importance of mental states conversation and the relevance of a dynamic approach to training and of the variability of the tasks used.

### Conflict of Interest Statement

The authors declare that the research was conducted in the absence of any commercial or financial relationships that could be construed as a potential conflict of interest.
